# The Ontario Integrated Supervised Injection Services Cohort Study of People Who Inject Drugs in Toronto, Canada (OiSIS-Toronto): Cohort Profile

**DOI:** 10.1007/s11524-021-00547-w

**Published:** 2021-06-28

**Authors:** Ayden I. Scheim, Ruby Sniderman, Ri Wang, Zachary Bouck, Elizabeth McLean, Kate Mason, Geoff Bardwell, Sanjana Mitra, Zoë R. Greenwald, Kednapa Thavorn, Gary Garber, Stefan D. Baral, Sean B. Rourke, Dan Werb

**Affiliations:** 1 Department of Epidemiology and Biostatistics, Dornsife School of Public Health, Drexel University, Philadelphia, PA USA; 2 Centre on Drug Policy Evaluation, Li Ka Shing Knowledge Institute, St. Michael’s Hospital, 30 Bond Street, Toronto, ON M5B 1X1 Canada; 3 Department of Epidemiology and Biostatistics, Schulich School of Medicine and Dentistry, Western University, London, ON Canada; 4 MAP Centre for Urban Health Solutions, Li Ka Shing Knowledge Institute, St. Michael’s Hospital, Toronto, ON Canada; 5 Dalla Lana School of Public Health, University of Toronto, Toronto, ON Canada; 6 South Riverdale Community Health Centre, Toronto, ON Canada; 7 Department of Medicine, University of British Columbia, St. Paul’s Hospital, 608-1081 Burrard Street, Vancouver, BC Canada; 8 British Columbia Centre on Substance Use, 400-1045 Howe Street, Vancouver, BC Canada; 9 Interdisciplinary Studies Graduate Program, University of British Columbia, 2357 Main Mall, Vancouver, BC 270, V6T 1Z4 Canada; 10 Ottawa Hospital Research Institute, The Ottawa Hospital, Ottawa, ON Canada; 11 School of Epidemiology and Public Health, University of Ottawa, Ottawa, ON Canada; 12 Department of Medicine, University of Ottawa, Ottawa Hospital Research Institute, 501 Smyth Box, Ottawa, ON Canada; 13 Department of Epidemiology, John Hopkins University School of Public Health, Baltimore, MD USA; 14 Department of Psychiatry, University of Toronto, Toronto, ON Canada; 15 Institute of Health Policy, Management and Evaluation, University of Toronto, Toronto, ON Canada; 16 Division of Infectious Diseases and Global Public Health, University of California San Diego School of Medicine, La Jolla, CA USA

**Keywords:** Cohort studies, People who inject drugs, Supervised injection, Health services research, Addictions

## Abstract

The Ontario Integrated Supervised Injection Services cohort in Toronto, Canada (OiSIS-Toronto) is an open prospective cohort of people who inject drugs (PWID). OiSIS-Toronto was established to evaluate the impacts of supervised consumption services (SCS) integrated within three community health agencies on health status and service use. The cohort includes PWID who do and do not use SCS, recruited via self-referral, snowball sampling, and community/street outreach. From 5 November 2018 to 19 March 2020, we enrolled 701 eligible PWID aged 18+ who lived in Toronto. Participants complete interviewer-administered questionnaires at baseline and semi-annually thereafter and are asked to consent to linkages with provincial healthcare administrative databases (90.2% consented; of whom 82.4% were successfully linked) and SCS client databases. At baseline, 86.5% of participants (64.0% cisgender men, median ([IQR] age= 39 [33–49]) had used SCS in the previous 6 months, of whom most (69.7%) used SCS for <75% of their injections. A majority (56.8%) injected daily, and approximately half (48.0%) reported fentanyl as their most frequently injected drug. As of 23 April 2021, 291 (41.5%) participants had returned for follow-up. Administrative and self-report data are being used to (1) evaluate the impact of integrated SCS on healthcare use, uptake of community health agency services, and health outcomes; (2) identify barriers and facilitators to SCS use; and (3) identify potential enhancements to SCS delivery. Nested sub-studies include evaluation of “safer opioid supply” programs and impacts of COVID-19.

## Background

It is estimated that 2016, 0.70% of Canadians aged 15 to 64 years used injection drugs, up from 0.55% in 2011 [1]. In the context of unregulated drug markets and unsafe injecting practices, injection drug use is associated with overdose, HIV and Hepatitis C transmission, soft tissue infections, and endocarditis [1]. Overdose rates in Canada have increased due to penetration of fentanyl and its analogues in the illicit drug market; 82% of accidental opioid overdose deaths in 2020 involved fentanyl [2]. In Ontario, Canada’s most populous province, there were 15.3 opioid-related overdose deaths per 100,000 people between January and September 2020, among the highest provincial rates in the country [2]. Toronto, Ontario’s capital, accounts for 20% of the province’s overdose deaths [3]. In response to an already-worsening opioid overdose crisis, supervised consumption services (SCS) in Toronto began to open in 2017, along with scale-up of other harm reduction interventions (e.g., naloxone distribution, opioid agonist treatment [OAT]).

SCS are health services where people who inject drugs (PWID) consume pre-obtained drugs under the supervision of health professionals and/or trained peers, and can access clean injection/inhalation equipment, basic healthcare such as wound care, and referrals to other services [4]. Many SCS also permit swallowing, snorting, and (less commonly in Canada) smoking of drugs. Staff are trained to respond to overdoses, and there are no recorded cases of overdose death within an SCS [5, 6]. There are approximately 120 SCS worldwide with operating models including stand-alone storefronts, injecting rooms integrated within existing community health services, in-hospital SCS, and mobile vans [5, 6].

Individual-level and ecological studies of SCS effectiveness have indicated that SCS are associated with reduced overdose mortality, reduced syringe sharing and infection disease risk, and increased uptake of detoxification and addiction treatment services [6–9]. SCS implementation has also been associated with decreases in public injecting and publicly discarded syringes [6, 10, 11]. In Vancouver, frequent SCS use was associated with reduced all-cause mortality among PWID [12]. There is no scientific evidence that SCS are associated with increases in injection drug use initiation or crime [6, 13, 14].

Existing quantitative evaluations of SCS have necessarily been observational or quasi-experimental, given the lack of equipoise to justify a randomized trial [14, 15]. However, potentially addressable limitations of the extant evidence have been identified, including a lack of SCS-unexposed comparison groups in most studies, assumptions about whether behavior change within SCS extends to off-site drug use, and limitations of self-report [15]. In addition, approximately 80% of existing SCS research focuses on two sites in Vancouver, Canada, and Sydney, Australia [6, 13, 14]. These sites have similar high-volume, stand-alone models and most of the studies were conducted prior to the current overdose crisis. Vancouver differs from other Canadian cities in multiple respects relevant to SCS implementation, including a dense concentration of injection drug use in one neighborhood and a temperate climate. Therefore, there is a critical need for studies that examine the effectiveness of SCS in a wider range of geographic and cultural settings, and in the context of heightened overdose risk. Furthermore, there have been calls for research on implementation specifics of SCS (e.g., design, [co]-location, service integration, rules, hours of operation) to understand how various features of SCS impact their effectiveness [14, 16].

To address these critical gaps, the Ontario Integrated Supervised Injection Services cohort study in Toronto (OiSIS-Toronto) was established in November 2018 to evaluate the first three sanctioned SCS in the city. Each is integrated within an existing community health agency (CHA), including two comprehensive community health centers and one harm reduction program. SCS were first implemented in Toronto in the summer of 2017 with an unsanctioned site situated in a downtown park, [17] and the first sanctioned SCS opened in August 2017. Both SCS and overdose prevention sites are herein referred to collectively as SCS, a term we use to reflect that the sites permit consumption by multiple routes. As of December 2020, there were nine SCS operating in Toronto. In response to the worsening overdose crisis, a range of harm reduction innovations emerged beyond SCS, notably a street drug-checking program [18] and “safer opioid supply (SOS)” programs. SOS are harm reduction programs aiming to reduce overdose risk by prescribing pharmaceutical opioids in place of unregulated street opioids [19]. These are interventions with high plausibility but limited empirical evidence bases; therefore, the cohort additionally provides an evaluative framework to test these emerging harm reduction interventions. Herein, we describe the cohort protocol and participant characteristics.

## Methods

### Study Goals and Design

OiSIS-Toronto is an open prospective cohort of PWID who do and do not use SCS. The primary goals of the OiSIS cohort are to (1) evaluate the impact of various integrated SCS models on client uptake of CHA programming, health service use, and health outcomes; (2) to identify barriers to SCS use; and (3) to identify potential operational and regulatory enhancements to SCS. The cohort draws on three data sources: (1) semi-annual interviewer-administered study questionnaires; (2) linkages to administrative databases at ICES, Ontario’s repository for vital statistics and health administrative data; and (3) linkages to visit-level data from electronic SCS databases at the three CHAs. Consent is sought separately for data linkages and participants may enroll in the cohort without consenting to linkage.

Baseline data collection began on 5 November 2018; as the cohort is open, herein we report on participants enrolled by 19 March 2020, prior to the temporary suspension of new enrolments due to COVID-19 restrictions in Toronto. At present, we plan to follow participants up to 2.5 years from baseline for a total of six study visits occurring at semi-annual intervals. The study was approved by the Research Ethics Boards at Unity Health Toronto, the University of Toronto, and Toronto Public Health.

### Setting

Toronto is the largest city in Canada (2017 population = 2.93 million) and among the most ethno-racially diverse in the world. Beginning in 2016, overdose rates in Toronto rose precipitously as the street opioid supply became increasingly potent, as heroin was supplemented with, and ultimately largely replaced by, fentanyl [4, 20]. The first three SCS in Toronto, which OiSIS-Toronto was established to evaluate, opened between August 2017 and March 2018. Characteristics of these SCS are described in Table 1.

**Table 1:** SCS participating in the OiSIS-Toronto cohort study

Characteristic	Queen West Community Health Centre	South Riverdale Community Health Centre	The Works
Service model	SCS integrated in health center providing medical, mental health, and social care to vulnerable populations	SCS integrated in health center providing medical, mental health, and social care to vulnerable populations	SCS integrated in harm reduction program serving persons who use drugs, including on-site nursing care and OAT clinic
Consumption modes	Injection; intranasal; oral (>99% injection)	Injection; intranasal; oral (>95% injection)	Injection
Peer-assisted injecting allowed? (as of March 2020)	Yes	Yes	No
Street drug checking service available?	Yes	Yes	Yes
Booths and hours of operation	Four booths; 45.5 h/week over 5 days	Four booths; 42.0 h/week over 5 days	Six booths, 78.0 h/week over 7 days
Average visits per month (until March 2020)	522	549	3200

### Participants

At baseline, eligible participants had to be 18 years of age or older, report injecting illicit drugs within the previous 6 months, able to speak and understand English, and residing in Toronto. Recruitment materials specified that participants did not need to use SCS to enroll. Clients of the study SCS were primarily recruited on-site at the CHAs. Recruitment of participants who do not use SCS was designed with the aim of composing a comparator group experiencing similar structural vulnerabilities as those who use SCS, and involved a combination of active outreach by study staff at CHAs; non-incentivized peer recruitment; and passive recruitment (posters, recruitment cards) at health and social service organizations, on the street, and in needle and syringe packages distributed by CHAs. To avoid eligibility restrictions that might lead to misrepresentation, we also enrolled individuals who use non-study SCS, using the same methods as for non-SCS users. These participants are grouped with other SCS users for analyses of overall SCS outcomes and disaggregated where particular SCS models are being compared. Study interviews are completed at one of the three participating CHAs, or at the research team offices if an alternative site is requested by a participant. At each visit, participants complete an interviewer-administered computer-based questionnaire and receive a CDN $30 honorarium. All participants provided written informed consent for cohort participation and (separately) data linkages.

#### Retention and Follow-Up

OiSIS-Toronto employs a number of retention strategies commonly used in PWID cohorts [21, 22]. Multiple forms of contact information were collected for each participant, including identifying two persons who know but do not live with the participant and for social service organization staff with whom they regularly interacted. Drop-in hours are advertised during which participants can sign up for a same-day interview. Participants can receive honoraria for checking in with study staff between interview visits (CDN $5 at the beginning of the study, later increased to $10). We also use reminder cards, posters, and a toll-free number so that participants may phone the study office long-distance or from jail.

### Data Sources

Variables to be derived from all data sources are summarized in Table 2.

**Table 2:** Variables and data sources available for OiSIS-Toronto participants at baseline

Content area	Key variables	Source
Demographics and socio-structural exposures	Age, sex assigned at birth and gender identity, sexual orientation, race/ethnicity, education, income and income sources, housing, recent incarceration	Questionnaire
Drug use behaviors	Injection and non-injection drug use (drugs used, frequency), fentanyl-related behaviors and attitudes, public injection, syringe and equipment sharing, provision of injection initiation assistance, alcohol use [AUDIT-C] [23]	Questionnaire
SCS use	SCS use, SCS-related behavior change, SCS satisfaction, SCS services accessed, referrals accessed	Questionnaire
Overdose and other health conditions	Overdose history and response, HIV status and treatment, Hepatitis C status and treatment, depression [PHQ-9], [24] drug-use related stigma	Questionnaire
Drug checking services (DCS)	Interest and actual use of various DCS technologies	Questionnaire
Substance use disorder treatment	Engagement in pharmacologic and non-pharmacologic treatment, satisfaction and preferences	Questionnaire
Healthcare use	Primary care, emergency department visits, and hospital admissions (timing, frequency, diagnoses); mortality and cause of death; dispensation of OAT	ICES
SCS visit data	Visit timing and frequency, drugs consumed, overdose events and disposition, referrals	SCS visit database

#### OiSIS-Toronto Questionnaire

The questionnaire includes self-reported data on demographic characteristics and socio-structural exposures, drug use behaviors, use of SCS, overdose experiences and other health conditions, use of drug checking services, and substance use disorder treatment.

#### Administrative Data

Cohort participants are asked to consent to a having their study questionnaire data linked with administrative data at ICES, which broadly captures demographic information, vital statistics, and publicly funded healthcare encounters in Ontario. Consenting participants provide their Ontario Health Insurance Plan (OHIP) number and/or one or more of their name, date of birth, and postal code. This information is then used to link consenting participants with ICES data holdings either via their OHIP number (deterministic linkage) or using probabilistic linkage methods. Questionnaire data will be linked with the following ICES databases: the Registered Persons Database (demographic and vital statistics for all OHIP-eligible residents); the OHIP claims database (billing covering ~95% of physicians in Ontario); the Discharge Abstract Database (hospital admissions and discharges); the National Ambulatory Care Reporting System (emergency department visits); the Client Agency Program Enrolment Registry (patient enrolment with individual primary care physicians); the Corporate Provider Database (physician and practice information); the Ontario Mental Health Reporting System (mental health admissions); CONTACT (eligibility summaries and yearly health services contact); the Ontario HIV Database (persons with HIV); and the Narcotic Monitoring System database (dispensations for controlled medications [including methadone and buprenorphine/naloxone], irrespective of payment method).

#### SCS Visit Database

Participating CHAs track SCS use in an electronic database; two of three sites use NEO 360, a computerized record-keeping system used by harm reduction programs across Ontario [25]. To track client-level visit data, clients are asked to create a unique identification code and to provide it at each visit but can opt-out of creating a code to access the service entirely anonymously. SCS use records are not linked to other CHA records, including electronic medical records. Participants who use the three study SCS are asked for permission to access their SCS visit history via their code.

### Nested Studies

#### Qualitative Studies of SCS Implementation Contexts

In the summer of 2018, a qualitative study was conducted at two study SCS to understand barriers and benefits to accessing integrated SCS, including any contextual factors that affected uptake [26, 27]. Individual semi-structured interviews were completed with 24 participants and ethnographic observations were gathered. In October 2019, rapid ethnographic research was conducted in the neighborhood surrounding a third SCS in downtown Toronto to examine public drug use and “public order.” This latter study included participant observation (e.g., police, security guards, people who use drugs, general public) and built environment observation, as well as informal conversations.

#### Quantitative Sub-studies

Since October 2020, OiSIS-Toronto participants who indicate they are enrolling in an SOS program have been invited to participate in a sub-study assessing clinical, health, and social outcomes associated with SOS. Additionally, we conducted a rapid survey to assess the impact of COVID-19 and related restrictions on people who use drugs in Canada, which was administered to 170 cohort participants between June 2020 and April 2021, using a combination of telephone and in-person interviewing, and which was also administered to cohorts of people who use drugs in Vancouver and Montreal.

### Community Engagement

Representatives of the CHAs participating in the study are co-investigators on the project. Participating CHAs have pre-existing community advisory bodies comprised of people who use drugs, who are compensated for their time and expertise. The community advisory bodies are consulted on an ongoing basis on study recruitment and retention procedures, priority research topics, and approaches for disseminating findings to community members. People with lived experience of drug use are prioritized for research staff positions; importantly, this commitment has required advocacy and creativity within a hospital system in which formal educational credentials and criminal background checks are typically required for hiring. While all staff eventually went through those institutional processes, we provided them with close support, including financial assistance to obtain identification needed for the background checks.

### Data Analysis

We calculated descriptive statistics to describe demographic, health status, and drug use characteristics of cohort participants, overall, and stratified by self-reported frequency of SCS use (use of SCS for none, few [≤25%], some [26-74%], or all or most [≥75%] of their injections) and by retention status (≥1 follow-up visit vs. lost to follow-up). We used Wilcoxon rank-sum tests and chi-square tests, for continuous and categorical variables respectively, to test for differences in characteristics. We also calculated frequencies of drug-related risks in the full sample.

## Results

### Recruitment and Data Linkage

**Fig. 1:**
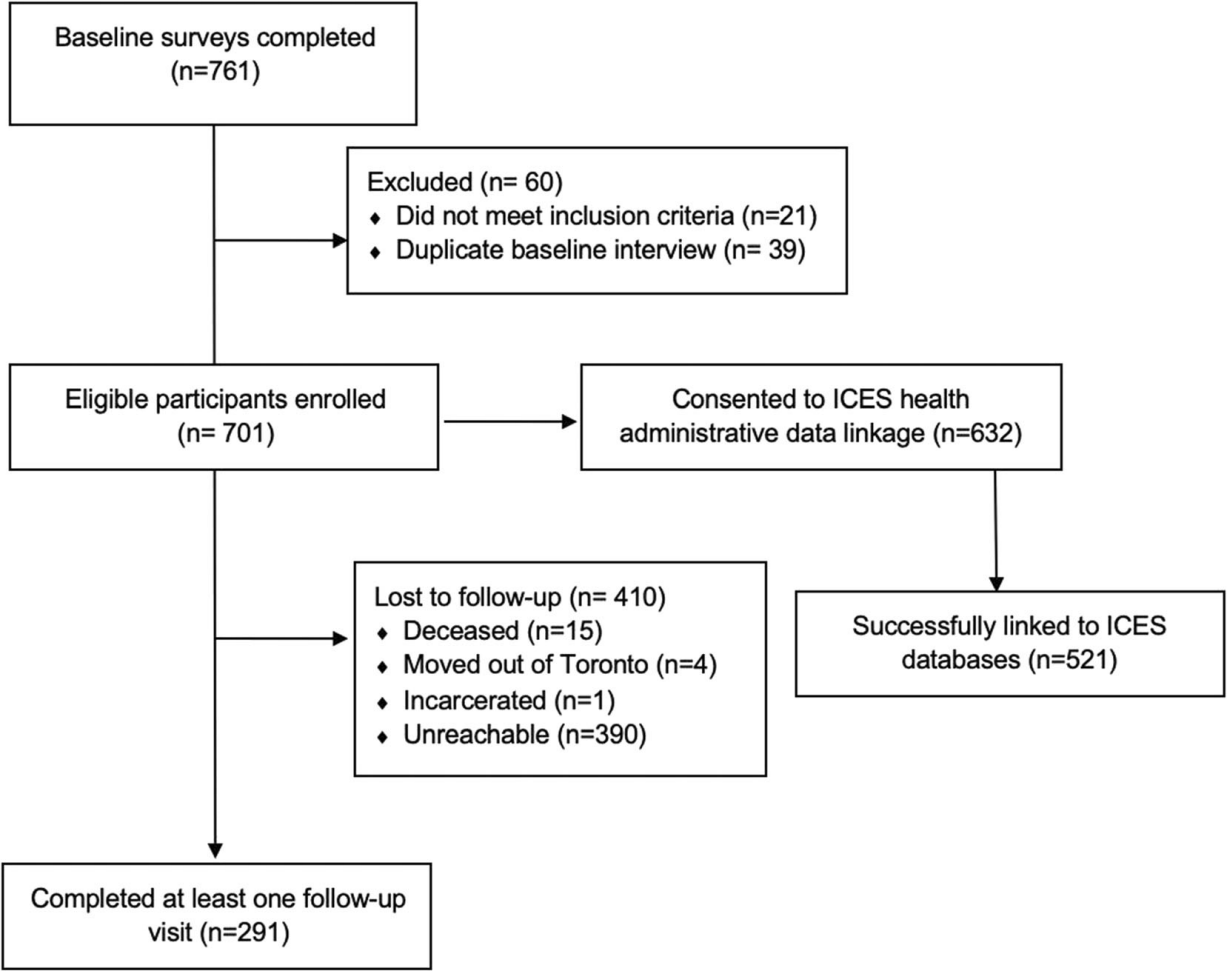
Participant flow

As shown in Fig. 1, as of 19 March 2020, 761 baseline interviews were conducted and 60 interviews were removed from the study due to ineligibility or duplicate interviews. In total, 701 eligible participants were enrolled, including 520 (74.2%) who used one of the SCS being evaluated, 89 (12.7%) who used other SCS, and 92 (13.1%) who did not use any SCS over the previous 6 months. As shown in Table 3, participating SCS clients were approximately evenly split between those who reported using SCS for all or most (≥75%; *n*=182), some (26–74%; *n*=215), or few (≤25%; *n*=204) of their injections.

**Table 3:** Characteristics of OiSIS-Toronto participants by recent frequency of SCS use

Characteristic	Total	Recent frequency of SCS use (proportion of all injections at SCS)*				*p* value
	*n*= 701 *n* (%)	All or most (≥75%) *n* = 182 *n* (%)	Some (26-74%) *n* = 215 *n* (%)	Few (≤25%) *n* = 204 *n* (%)	None *n* = 94 *n* (%)	
Age (med, [IQR])	39.0 (33.0–49.0)	39.0 (33.0–48.0)	36.0 (30.5–44.0)	42.0 (34.0–51.0)	47.5 (37.3–52.8)	<0.01
Gender (*n*=698)						0.15
Cisgender man	449 (64.3)	105 (58.0)	140 (65.4)	132 (65.0)	66 (70.2)	
Cisgender woman	216 (30.9)	69 (38.1)	66 (30.8)	60 (29.6)	21 (22.3)	
Transgender or gender diverse	33 (4.7)	7 (3.9)	8 (3.7)	11 (5.4)	7 (7.4)	
Sexual orientation (*n*=695)						0.42
Sexual minority	129 (18.6)	31 (17.2)	34 (15.8)	43 (21.3)	20 (21.5)	
Straight or heterosexual	566 (81.4)	149 (82.8)	181 (84.2)	159 (78.7)	73 (78.5)	
Ethnoracial group (*n*=700)						0.91
Indigenous	235 (33.6)	59 (32.6)	70 (32.6)	71 (34.8)	33 (35.1)	
Racialized, non-Indigenous	90 (12.9)	20 (11.0)	27 (12.6)	29 (14.2)	14 (14.9)	
White, non-Indigenous	375 (53.6)	102 (56.4)	118 (54.9)	104 (51.0)	47 (50.0)	
Homeless or unstably housed^†^ (*n*=650)	588 (90.5)	154 (92.2)	185 (93.0)	170 (90.4)	75 (83.3)	0.06
Recent incarceration^†^ (*n*=650)	247 (38.0)	54 (32.3)	84 (42.2)	74 (39.4)	34 (37.8)	0.27
Income sources^†^ (*n*=701)						
Paid employment	116 (16.5)	24 (13.2)	36 (16.7)	36 (17.6)	18 (19.1)	0.54
Recycling	225 (32.1)	52 (28.6)	69 (32.1)	71 (34.8)	30 (31.9)	0.63
Panhandling	243 (34.7)	66 (36.3)	87 (40.5)	67 (32.8)	21 (22.3)	0.02
Government benefits	649 (92.6)	169 (92.9)	200 (93.0)	192 (94.1)	83 (88.3)	0.34
Friends and/or family	341 (48.6)	82 (45.1)	120 (55.8)	105 (51.5)	31 (33.0)	<0.01
Theft	344 (49.1)	94 (51.6)	115 (53.5)	101 (49.5)	32 (34.0)	0.01
Selling needles	18 (2.6)	4 (2.2)	5 (2.3)	7 (3.4)	2 (2.1)	0.84
Selling drugs	353 (50.4)	95 (52.2)	105 (48.8)	115 (56.4)	35 (37.2)	0.02
Sex work or transactional sex	120 (17.1)	36 (19.8)	42 (19.5)	27 (13.2)	13 (13.8)	0.20
Result of last HIV test (*n*=690)						<0.01
Never tested or did not receive results	41 (5.9)	9 (5.0)	15 (7.2)	10 (4.9)	7 (7.5)	
Positive	35 (5.1)	6 (3.3)	4 (1.9)	12 (5.9)	12 (12.9)	
Negative	614 (89.0)	165 (91.7)	189 (90.9)	181 (89.2)	74 (79.6)	
Ever diagnosed with Hepatitis C (*n*=641)	335 (52.3)	106 (61.3)	86 (44.3)	106 (57.0)	34 (41.5)	<0.01
Currently Hepatitis C-positive^‡^ (*n*=335)						0.56
Yes	191 (57.0)	57 (54.3)	54 (62.8)	61 (57.0)	17 (50.0)	
No	132 (39.4)	46 (43.8)	29 (33.7)	40 (37.4)	16 (47.1)	
Do not know	12 (3.6)	2 (1.9)	3 (3.5)	6 (5.6)	1 (2.9)	
Drug injected most often (*n*=693)						<0.01
Fentanyl	333 (48.1)	99 (54.7)	134 (63.2)	78 (38.6)	19 (20.7)	
Heroin	58 (8.4)	16 (8.8)	10 (4.7)	21 (10.4)	11 (12.0)	
Prescription opioids (e.g., hydromorphone, morphine)	56 (8.1)	15 (8.3)	11 (5.2)	25 (12.4)	4 (4.3)	
Crystal methamphetamine	137 (19.8)	26 (14.4)	35 (16.5)	46 (22.8)	28 (30.4)	
Cocaine or crack/rock cocaine	86 (12.4)	15 (8.3)	17 (8.0)	26 (12.9)	28 (30.4)	
Other	23 (3.3)	10 (5.5)	5 (2.4)	6 (3.0)	2 (2.2)	
Number of years injecting (median, IQR) (*n*=691)	13.0 (6.0–26.0)	13.0 (5.0–24.3)	11.0 (5.0–18.0)	17.0 (7.0–31.0)	18.0 (8.0–31.5)	<0.01
Current use of opioid agonist therapy (*n*=699)						0.01
No	472 (67.5)	107 (58.8)	142 (66.4)	147 (72.1)	72 (77.4)	
Yes—methadone	188 (26.9)	67 (36.8)	59 (27.6)	43 (21.1)	18 (19.4)	
Yes—buprenorphine/naloxone	34 (4.9)	6 (3.3)	13 (6.1)	11 (5.4)	3 (3.2)	
Yes—other	5 (0.7)	2 (0.1)	0 (0.0)	3 (1.5)	0 (0.0)	
Total	--	182 (26.2)	215 (30.9)	204 (29.4)	94 (13.5)	--

^*^Six participants were missing data on frequency of SCS use in the past 6 months

^†^In the previous 6 months

^‡^Among those ever diagnosed

Note: proportions may not sum to 100% because of rounding

*IQR* interquartile range, *SCS* supervised consumption site

Overall, 632 participants (90.2%) consented to healthcare administrative data linkages and 521 (82.4%) were successfully linked to ICES databases. In total, 280 study SCS clients (53.8%) provided their client identification code for SCS database linkage, with substantial variation across sites (range = 28–66%), likely due to variation in the extent to which sites actively promote creation and use of a consistent code.

### Socio-Demographic Characteristics

Socio-demographic characteristics and drug use patterns are shown in Table 3, stratified by frequency of SCS use. At baseline, the median age of participants was 39 (interquartile range [IQR]: 33–49), and two-thirds were cisgender men (64.3%, *n*=449). Approximately half of the participants were white (53.6%) and one-third were Indigenous (33.6%, *n*=235). The vast majority of participants were homeless or unstably housed (90.7%, *n*=584/650), and over one-third had recently been incarcerated (38.0%, *n*=247/650). Most socio-demographic characteristics did not vary significantly by frequency of SCS use; however, compared to SCS clients, non-SCS users were older (median [IQR] age = 47.5 [37.3–52.8]) and less likely to report some illicit or informal income sources.

### HIV and Hepatitis C

Five percent of participants self-reported being HIV-positive (*n*=35); self-reported HIV prevalence was lower among those who used SCS more frequently (*p* <0.01). More than half (52.3%, *n*=335) reported a lifetime diagnosis of Hepatitis C, of whom 57% (*n*=191) reported current, chronic infection.

### Drug Use and Treatment Patterns

Approximately half of participants (48.1%, *n*=333) reported fentanyl as their most frequently injected drug and SCS clients were more likely to do so (*p* <0.01). Among non-SCS users, stimulant use was most common, with 60.8% (*n*=56) injecting either crystal methamphetamine or cocaine most often. Participants had been injecting for a median of 13 years; those who used SCS more frequently had shorter injecting histories (*p* <0.01). Almost one-third (32.5%, *n*=227) were receiving OAT at the time of their interview, including methadone (26.9%, *n*=188) and buprenorphine/naloxone (4.9%, *n*=34). OAT use increased with SCS use frequency (*p*=0.01).

### Drug-Related Risks

Drug-related risks in the full sample are shown in Table 4. Over half of participants reported injecting daily (56.8%, *n*=398) over the previous 6 months and the majority (81.4%, *n*=570) reported injecting in public. Most had injected alone (88.1%, *n*=614) and 41.3% (*n*=289) had needed help to inject. Most participants did not share syringes (14.9% borrowed and 17.1% lent; *n*=104 and 120) but more commonly reported sharing other injecting equipment such as cookers or filters (37.2%, *n*=260). The majority of participants (70.0%, *n*=489) had ever overdosed, and 38.6% (*n*=270) reported an overdose within the previous 6 months. In a published analysis, we found that this overdose risk was not independently associated with frequency of SCS use [28].

**Table 4:** Drug-related risks among OiSIS-Toronto participants

	Total *n* (%)
Frequency of injection* (*n*=701)	
Daily	398 (56.8%)
More than once a week	158 (22.5%)
Once a week	41 (5.8%)
Less than weekly	104 (14.8%)
Injected in a public place* (*n*=700)	
Always or most of the time	108 (15.4%)
Some of the time or occasionally	462 (66.0%)
Never	130 (18.6%)
Injected alone* (*n*=697)	
Always or most of the time	183 (26.3%)
Some of the time or occasionally	431 (61.8%)
Never	83 (11.9%)
Needed help to inject* (*n*=701)	
Always or most of the time	73 (10.4%)
Some of the time or occasionally	216 (30.9%)
Never	411 (58.7%)
Borrowed a used syringe* (*n*=698)	104 (14.9%)
Lent a used syringe* (*n*=701)	120 (17.1%)
Receptively shared other injecting equipment* (*n*=699)	260 (37.2%)
Filled syringe from a syringe used by someone else* (*n*=696)	141 (20.3%)
Smoked crack cocaine* (*n*=562)	
Daily or more than once a week	205 (36.5%)
Weekly to monthly	99 (17.6%)
Less than monthly	53 (9.4%)
Never	205 (36.5%)
Smoked crystal methamphetamine* (*n*=565)	
Daily or more than once a week	113 (20.0%)
Weekly to monthly	102 (18.1%)
Less than monthly	51 (9.0%)
Never	299 (52.9%)
Heavy alcohol use (≥6 drinks in one sitting)* (*n*=698)	
Daily or more than once a week	111 (15.9%)
Weekly to monthly	86 (12.3%)
Less than monthly	104 (14.9%)
Never	397 (56.9%)
Ever overdosed (*n*=699)	489 (70.0%)
Recent overdose* (*n*=699)	270 (38.6%)

*Over the previous 6 months

### Retention

As of 23 April 2021, 291 participants (41.5%) had completed at least one follow-up visit. Of the 410 (58.5%) deemed lost to follow-up, 15 were known to have deceased, one was known to be incarcerated, four had moved away, and 390 were unreachable. Participants lost to survey follow-up will remain included in administrative data analysis. Baseline SCS use frequency varied by retention status. Specifically, those lost to follow-up were more likely to be non-SCS-users (16.5%, *n*=67 of those lost to follow-up vs. 9.3%, *n*=27 of those retained) and less likely to use SCS for all/most injections (23.4%, *n*=95 vs. 30.1%, *n*=87); these differences were statistically significant (*p*=0.03). Participants lost to follow-up were also less likely to report income from selling drugs (45.6%, *n*=187 vs. 57.0%, *n*=166, *p*<0.01) at baseline. For all other variables in Table 3, there were no significant differences between participants based on retention status (data not shown).

### Qualitative Studies

In published findings from qualitative interviews with 24 clients from two SCS integrated within community health centers, participants identified benefits of the integrated SCS model including convenience and access to other health and social services [26]. Challenges of the model included limited hours of operation and building design, which were perceived as compromising participants’ privacy and anonymity. Additionally, an analysis of policing in relation to SCS access found site-specific perceptions and experiences related to differences in local drug scenes, neighborhood contexts, and police practices [27]. Interviews and observations from one site emphasized a heightened police presence in the surrounding area that led to fears of harassment and arrest whereas there was a lack of police presence at the other site, and consequently minimal fear of police encounters or potential arrest when accessing SCS.

## Discussion

Longitudinal studies evaluating impacts of SCS in Canada are almost exclusively based on the Insite facility in Vancouver, Canada [6, 14]. Although the observational evaluation of Insite demonstrated numerous benefits, further research is needed to understand the impacts of diverse SCS models, in a range of geographic settings, and in the context of the current opioid overdose and emerging COVID-19 public health emergencies [14, 16]. To address these gaps, the OiSIS-Toronto cohort has recruited 701 PWID and will follow their health and social outcomes using a combination of self-report and administrative data. As indicated by the baseline data presented herein, and consistent with previous studies of SCS clients, [29] the study population experiences multiple forms of social and structural vulnerability: over 9 in 10 were homeless or unstably housed and over one-third were recently incarcerated. Almost half are of Indigenous ancestry and/or belong to racialized groups that, as a consequence of structural racism, often experience poorer health in Canada [30]. About half of participants reported that fentanyl was their most commonly used drug and consequently the study population is at high risk of overdose. Over a third reported having overdosed in the previous 6 months. This is comparable to the estimated lifetime prevalence of overdose among PWID globally, [31] attesting to the greater risk presented by unpredictable concentrations of fentanyl and other potent synthetic opioids present in Toronto’s street drug supply [20]. Current OAT enrollment (32.5%) was low, indicating an unmet need for patient-centered treatment in this population at high overdose risk.

Future analyses using the OiSIS-Toronto data will assess how SCS use, including use of various SCS models, relates to self-reported behaviors as well as OAT and other healthcare use ascertained through health administrative data linkages. A notable strength of the cohort is the use of administrative data to objectively ascertain utilization of publicly insured primary and hospital care, as well as OAT dispensation. Few previous SCS evaluations have included healthcare data linkages, and only to a single local hospital [32] or to local detoxification facilities [33]. It is notable that the vast majority of participants (90.2%) consented to administrative data linkages (for which no additional incentive was provided), of whom most (82.4%) were successfully linked, despite the majority of consenting participants having not provided complete information. Furthermore, access to the SCS visit database to validate self-reported frequency of SCS use will facilitate the application of bias-adjustment methods.

An additional strength of the cohort lies in the opportunity to evaluate the uptake and effectiveness of emergent harm reduction interventions using quantitative, qualitative, and mixed methods, including legally sanctioned drug checking services and safer opioid supply programs.

These programs are being implemented by the CHAs where our study SCS are located, allowing for a rapid research response, including embedding survey questions on service preferences, facilitators and barriers to uptake, and client experiences.

Recruiting and retaining socially and structurally vulnerable PWID in longitudinal research are challenging, [21, 34, 35] and OiSIS-Toronto has faced limitations in this regard, even prior to the COVID-19 pandemic. First, recruitment of PWID who do not use SCS was slower than anticipated despite employing multiple means of outreach and referral. This may reflect that a high proportion of PWID in Toronto has already been exposed to SCS, as well as the lack of a dense geographic concentration of PWID. This is compounded by resource limitations precluding the placement of study outreach workers at multiple locations. In contrast, most Canadian longitudinal research with PWID comes from Vancouver, Canada, where PWID, SCS, and cohort study offices are concentrated in one compact area and PWID are highly familiar with longitudinal cohort studies [36].

Similar factors may have impacted study retention. Due to high rates of homelessness, incarceration, and mortality, loss to follow-up is typically high in PWID cohorts, ranging from 20 to 50% per year [21, 35, 37, 38]. Our loss to follow-up is 58.5%, primarily because of the inability to reach participants through any of the means of contact they provided. Considering the high unconfirmed mortality rate of 2.1%, based on word of mouth alone, it is likely that our administrative data linkage will reveal that a higher proportion of loss to follow-up was related to participant death. Incarceration cannot be ascertained through currently available data linkages but likely contributes significantly to the inability to reach participants. In addition to the aforementioned factors impacting retention, the COVID-19 pandemic has substantially impacted our capacity to recruit and retain participants, and this impact is ongoing. Innovative retention strategies increasingly used in cohort studies, such as use of social media to keep in touch with participants and reminders from CHA staff and clinicians, [41–41] have been considered by the research team to enhance retention. However, we encountered challenges in engaging with a research ethics board with experience in in-hospital clinical studies and continue to work to adapt ethics processes to community-based research designs.

Although there were few observed differences between participants lost to follow-up and those retained for at least one visit, retention of participants who did not use SCS was poorer due to limited opportunities for opportunistic engagement with field study staff. Ongoing efforts will focus on recruitment and retention of non-SCS-users in close geographic proximity to SCS sites. We note that the inclusion of non-SCS comparison groups presents challenges across studies due to issues of confounding by indication [12, 28]. Future research in other settings should consider prospectively recruiting PWID prior to SCS implementation; this was the original intended design of OiSIS-Toronto but was not possible due to the timing of study funding and earlier-than-expected opening of SCS.

Finally, non-random sampling is an important limitation to generalizability of our study but typical of PWID cohorts. Random selection of SCS clients was not possible due to the operational features of the study sites (e.g., allowing for anonymity); however, we recruited a large fraction of SCS clients (the three CHAs had an estimated total of 989 active unique clients over the quarter preceding March 2020).

## Conclusions

With a focus on evaluating the secondary impacts of SCS on healthcare use and identifying how service integration and other operational characteristics impact client outcomes, the OiSIS-Toronto cohort responds to the need for a “second generation” of SCS research that examines how heterogeneous models and client populations impact SCS effectiveness [16]. The cohort further provides the opportunity to evaluate a number of emergent interventions being delivered within Toronto’s network of harm reduction services as well as the impact of COVID-19 on the health, social well-being, and clinical trajectories of PWID. Ultimately, administrative data linkages with very high opt-in will enable retrospective and prospective studies of the impact of access to integrated SCS models on healthcare and OAT outcomes.
